# Network flexibility facilitates treatment-induced recovery in post-stroke aphasia

**DOI:** 10.1093/braincomms/fcag279

**Published:** 2026-09-18

**Authors:** Isaac Falconer, Maria Varkanitsa, Anne Billot, Swathi Kiran

**Affiliations:** Chobanian & Avedisian School of Medicine, Boston University, Boston, MA 02118, USA; Center for Brain Recovery, Boston University, Boston, MA 02215, USA; Center for Brain Recovery, Boston University, Boston, MA 02215, USA; Department of Speech, Language, and Hearing Sciences, Sargent College of Health and Rehabilitation Sciences, Boston University, Boston, MA 02215, USA; Department of Neurology, Massachusetts General Hospital and Harvard Medical School, Charlestown, MA 02115, USA; Department of Psychology, Center for Brain Science, Harvard University, Cambridge, MA 02138, USA; Center for Brain Recovery, Boston University, Boston, MA 02215, USA; Department of Speech, Language, and Hearing Sciences, Sargent College of Health and Rehabilitation Sciences, Boston University, Boston, MA 02215, USA

**Keywords:** aphasia, stroke, dynamic functional connectivity, functional MRI

## Abstract

Predicting the recovery of post-stroke aphasia, particularly in the chronic phase, poses significant challenges. While many studies have focused on static resting state functional connectivity measures as biomarkers for language recovery, there is a significant lack of studies investigating dynamic functional connectivity, which takes into account second to minute timescale variation in connectivity and has been used extensively in other contexts. This retrospective cohort study investigates the predictive value of dynamic functional connectivity for treatment response in post-stroke aphasia. Two temporal metrics, temporal variability and community stability were explored. Temporal variability quantifies the magnitude of variation in the strength of functional connections over time, while community stability measures the mean length of time over which dynamic functional connectivity remains relatively stable. Additionally, a dynamic functional connectivity states analysis, in which time windows are clustered into states using a k-means clustering algorithm, was used to investigate time-varying network properties of dynamic functional connectivity in relation to treatment response. Baseline MRI data were collected for 30 participants with chronic post-stroke aphasia who received a semantic treatment aimed at improving word-finding. Dynamic functional connectivity was generated using a sliding-window approach and the relationships between treatment-induced improvements in naming accuracy and the respective temporal metrics and network properties were evaluated using linear mixed effects models predicting naming accuracy. Specifically, a significant interaction effect of session (i.e. time) and the respective measure was interpreted as a significant relationship between the measure and treatment response. Both temporal metrics were found to be significantly associated with naming improvements, with both higher temporal variability (i.e. magnitude of fluctuations in dynamic functional connectivity; interaction effect: *β* = 0.45, *P* = 3.6e−09, *η*1 = 0.072) and higher community stability (i.e. moment-to-moment stability of dynamic functional connectivity; interaction effect: *β* = 0.0046, *P* = 5.4e−06, *η*^1^ = 0.044) predicting greater treatment gains. These findings suggest that patients who benefit most from treatment have neural dynamics characterized by a large magnitude, but low frequency, of fluctuations in network synchrony. Finally, consistent with previous studies, patients who spent more time in a state characterized by higher modularity showed greater treatment response (interaction effect: *β* = 7.9e−05, *P* = 6.2e−04, *η*^1^ = 0.025). Overall, the findings of this study provide evidence for the utility of dynamic functional connectivity in studying post-stroke language recovery as a measure that captures interindividual difference in the capacity for recovery. They create a new direction for post-stroke aphasia research focusing on temporal dynamics and their relationship to long-term functional network plasticity.

## Teaser

Falconer *et al*. report that dynamic functional connectivity metrics are predictive of treatment response in post-stroke aphasia with higher temporal variability, greater community stability, and more time spent in a high-modularity state predicting greater gains. These findings highlight dynamic functional connectivity as a promising biomarker for language recovery potential.

## Graphical Abstract

**Graphical Abstract:**
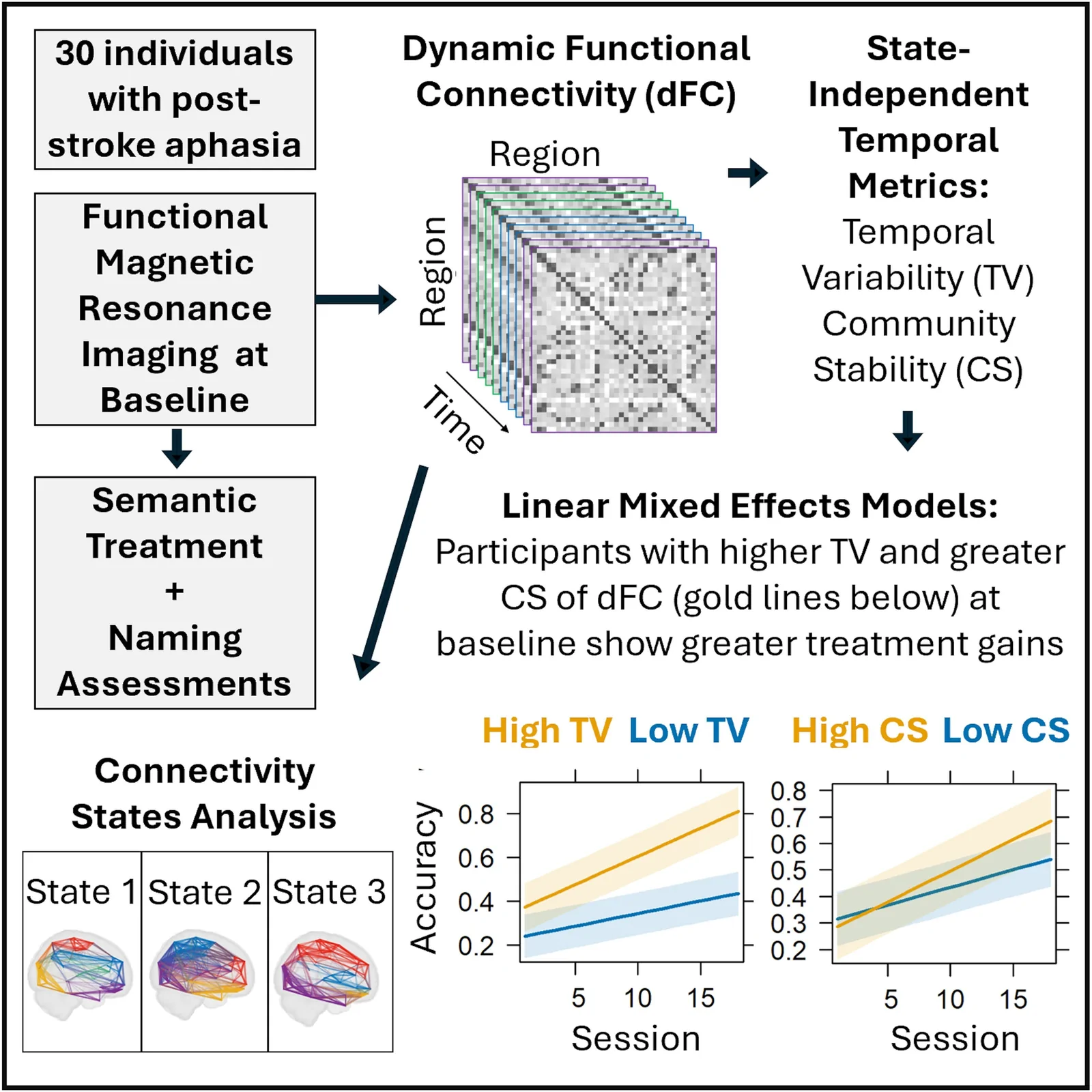

## Introduction

Aphasia, a common and devastating consequence of stroke, is a language disorder that affects the ability to speak, understand, read and write. Post-stroke aphasia (PSA) occurs in about one-third of stroke survivors, and often leads to significant disability and reduced quality of life.^1,2^ Predicting recovery is challenging due to significant individual variability, driven by multiple factors, including aphasia type, lesion-related factors such as size and location, structural imaging measures, and treatment-related factors such as type and duration, as well as interactions between those factors (e.g. aphasia type and treatment type).^3-11^ More recently, functional neuroimaging markers have improved prediction of PSA recovery, reflecting the complex interplay between local damage and large-scale network dysfunction.^9,12-17^

Resting-state functional connectivity (rsFC) is widely-used to study brain organization by examining intrinsic activity correlations in the absence of a task. While most rsFC studies on PSA have focused on static connectivity, averaging inter-regional coactivation over the entire course of a scan,^18-25^ dynamic functional connectivity (dFC) captures time-varying fluctuations in network organization. This approach offers a more detailed view of brain dynamics, revealing transient configurations that are lost in static analyses.^26-33^ dFC analyses often focus on two main types of analyses: (ⅰ) state-based analysis, which identifies recurring patterns of connectivity and quantifies metrics such as mean dwell time and state transitions^30,33-41^ and (ⅱ) state-independent temporal metrics such as temporal variability (TV), a measure of the magnitude of fluctuations in connectivity strength over time.^29,41^ Another temporal metric introduced here, community stability (CS), captures how long functional networks remain in a stable configuration before transitioning to a new state. These temporal metrics offer complementary insights into brain function. TV reflects network flexibility, with higher values reflecting a greater capacity of the brain to reshape its functional networks into diverse connectivity configurations. CS, on the other hand, provides a measure of network stability, which is thought to be important for maintaining coherent functional integration and may support cognitive processes like learning and recovery.^42-45^ State-based analysis offers additional context by revealing modular configurations of brain networks and their relevance to behaviour and recovery trajectories.^34-36^

Very few studies have investigated dFC in the context of PSA. Duncan and Small^46^ performed dFC analyses on resting state functional MRI data obtained before and after an imitation-based aphasia therapy. They found that improvement on a narrative task was positively associated with increases in time spent in a highly modular state (i.e. a state with relatively strong within-network connectivity and relatively weak between-network connectivity). This is consistent with static rsFC studies that have associated increases in modularity with stroke recovery and/or greater treatment gains.^46-48^ More recently, our group employed both state-based methods and a TV analysis to compare dFC in patients with PSA and healthy controls (Falconer, Varkanitsa, Billot, *et al*., in preparation). Patients were found to have reduced TV in spared language regions and their right hemisphere homotopes relative to healthy controls. Additionally, higher mean TV was associated with less severe aphasia, particularly in patients with larger lesions, suggesting that TV may be reflective of an individual’s capacity for language recovery after stroke. Results of the state-based analyses were consistent with Duncan and Small’s finding, with patients spending more time in a state characterized by low modularity compared with healthy controls. Additionally, this state had increased overall connectivity, particularly in the right hemisphere, which may reflect increased engagement of right hemisphere regions homotopic to damaged left hemisphere language regions as has been observed in previous studies.^49-52^

While dFC studies on PSA remain limited, several studies have investigated dFC in stroke recovery more broadly. Yue *et al*.^38^ for example, found that patients with post-stroke cognitive impairment had higher mean dwell times (i.e. spent more time) than healthy controls in a state characterized by relatively sparse connectivity. Additionally, mean dwell times in this state showed a significant negative correlation with scores on the Mini-Mental State Examination (MMSE) suggesting that the connectivity patterns that characterize this state represent a unique abnormality and a potential biomarker for post-stroke cognitive impairment. In motor recovery, Bonkhoff *et al*.^53^ identified a highly integrated state (i.e. increased synchronization between functional domains) for which higher dwell times were associated with poorer recovery, again supporting the importance of modular functional networks in stroke recovery.

Relatively few studies have examined temporal metrics of dFC in relation to longitudinal stroke recovery. Hu *et al*.^54^ observed lower TV of dFC in acute, subacute, and early chronic stroke patients compared with healthy controls and found gradual increases in the TV during stroke recovery. They also found that increased TV in the ipsilesional precentral gyrus from the acute to early chronic stage was associated with better motor recovery. In contrast, Chen *et al*.^55^ found higher TV of dFC in subacute stroke patients compared with healthy controls. Noting the difference in findings, the authors pointed to differences in samples between the two studies (e.g. stroke severity and lesion location). Notably, the latter study included haemorrhagic strokes, which were excluded from the former.

To summarize, in contrast to the relative wealth of literature on dFC in other neurological disorders, only a few studies have examined dFC in stroke recovery, with even fewer focusing on PSA. Building on our previous findings that TV is relevant to PSA, along with evidence linking TV and moment-to-moment network stability to learning and cognitive function,^42,43,45,54,56^ this study^61,44–47^ tests the hypothesis that greater overall flexibility in network configuration, combined with moment-to-moment temporal stability, supports functional reorganization underlying recovery. To test this hypothesis, we examined two temporal metrics of dFC in 30 patients with PSA before receiving aphasia therapy aimed at improving word-finding, namely TV and CS. Based on the hypothesis that higher TV reflects greater network flexibility facilitating functional reorganization, we predicted that higher baseline TV would be associated with greater rates of improvement in naming accuracy as a function of treatment due to enhanced network flexibility. Similarly, based on previous studies showing that network stability is related to better learning and overall cognitive ability,^42-45^ we hypothesized that greater CS supports productive engagement in treatment activities and thus predicted that greater CS would also be associated with greater improvements in naming accuracy. In addition to the analyses of dFC temporal properties, a dFC states analysis similar to Duncan and Small^46^ was performed. Based on Duncan and Small’s results and others,^46,48,57,58^ we hypothesized that segregation of locally specialized groups of regions reflects better network function and thus predicted that more time spent in higher modularity states would be associated with better treatment response.

## Materials and methods

### Participants

The study included 30 monolingual English-speaking individuals with aphasia due to a single left-hemisphere stroke in the chronic stage of recovery. The Western Aphasia Battery-Revised Aphasia Quotient (WAB AQ) was used to establish aphasia diagnoses and serve as an index of baseline aphasia severity. Aphasia therapy consisted of up to 18 2-h sessions of a typicality-based semantic treatment that was administered either two or three times per week. Treatment was discontinued if a criterion of 90% accuracy was reached for naming of trained items. Thirty-six items were trained for each participant, each of which was probed weekly. Each item fell into one of five categories, three of which were animate categories (birds, vegetables and fruits) and two of which were inanimate categories (i.e. clothing and furniture). They were balanced on lexical frequency, concreteness and familiarity. The naming percent accuracy for each session was used in statistical analyses. Additional details of the treatment protocol,^59^ the psycholinguistic properties of the trained items,^60^ and the behavioural^61^ and neuroimaging findings^9,62-66^ associated with this cohort of participants have been reported elsewhere. The study protocol was approved by Boston University’s Institutional Review Board and all participants provided written informed consent prior to study participation.

### MRI acquisition and preprocessing

MRI data were collected as part of a broader project, the Centre for the Neurobiology of Language Recovery (NIDCD P50DC012283), and these methods, including MRI acquisition, preprocessing, and lesion mapping, are also reported elsewhere.^9,25,59^ Images were acquired using Siemens 3.0T scanners. A 3D T1-weighted sequence was used to acquire structural images (TR/TE = 2300/2.98 ms, TI = 900 ms, flip angle = 9°, Field of View (FOV) = 256 × 256 mm^2^, voxel size = 1 × 1 × 1mm^3^, 176 sagittal slices with a slice thickness of 1 mm). A gradient-echo T2*-weighted sequence was used to acquire whole brain resting state functional scans (TR = 2.4 s, TE = 20 ms, flip angle = 90°, voxel size = 1.72 × 1.72 × 3 mm^3^, 210/175 interleaved slices). While the duration of scans varied between participants, all scans were truncated prior to dFC analyses such that 7 min of resting state data was included for all participants. During the scan, participants were instructed to allow their minds to wander and to stay awake with their eyes open.

Lesion maps were drawn manually by trained operators using MRIcron (http://www.sph.sc.edu/comd/rorden/mricron). The SPM Clinical Toolbox^67^ was used for enantiomorphic replacement of lesioned tissue in structural images before preprocessing. This step creates a pseudo-healthy scan by filling in lesioned areas using information from the healthy hemisphere, which reduces distortion effects from the lesion during normalization. After skull-stripping, the data were preprocessed in the Nipype-based tool fMRIPrep version 1.3.1^68^ using the N4 bias field correction algorithm (version 2.1.011)^69^ to correct structural volumes for intensity non-uniformity. Scans were normalized to the ICBM 152 Non-linear Asymmetrical template version 2009c using non-linear registration (ANTs version 2.1.0.). Segmentation of cerebrospinal fluid (CSF), white matter, and grey matter was performed using fast (FSL version 5.0.9) and mcflirt (FSL version 5.0.9) was used for motion correction of functional data. The motion-corrected volumes were then co-registered to T1 volumes with flirt (FSL) using boundary-based registration with nine degrees of freedom. Lesion masks were transformed to MNI space using the same non-linear deformation fields derived from structural image normalization. Voxels in the functional data that fell within the normalized lesion maps were set to zero using AFNI’s 3dcalc (v19.0.17).^70^ Lesion volume for each participant was measured as the number of voxels that fell within their lesion map (i.e. the sum of the lesion map binary 3D matrix).

The CONN Toolbox^71^ was used for additional preprocessing as well as to generate mean BOLD timeseries per ROI. Motion outliers in the functional volumes were detected and discarded with the Artifact Detection Tool using a composite motion threshold of 0.9 mm and a global signal threshold of 5 SDs from the mean. High pass filtering with a threshold of 0.01 Hz and regression of realignment parameters and white matter and CSF signal principal components (five principal components each) were used to remove motion, physiological noise and other artefacts. Mean BOLD timeseries per ROI, using the Animated Anatomical Labelling Atlas (AAL3),^72^ were then generated from resulting preprocessed functional data and were used in the subsequent dynamic functional connectivity (dFC) analyses.

### Dynamic functional connectivity

**Figure 1:**
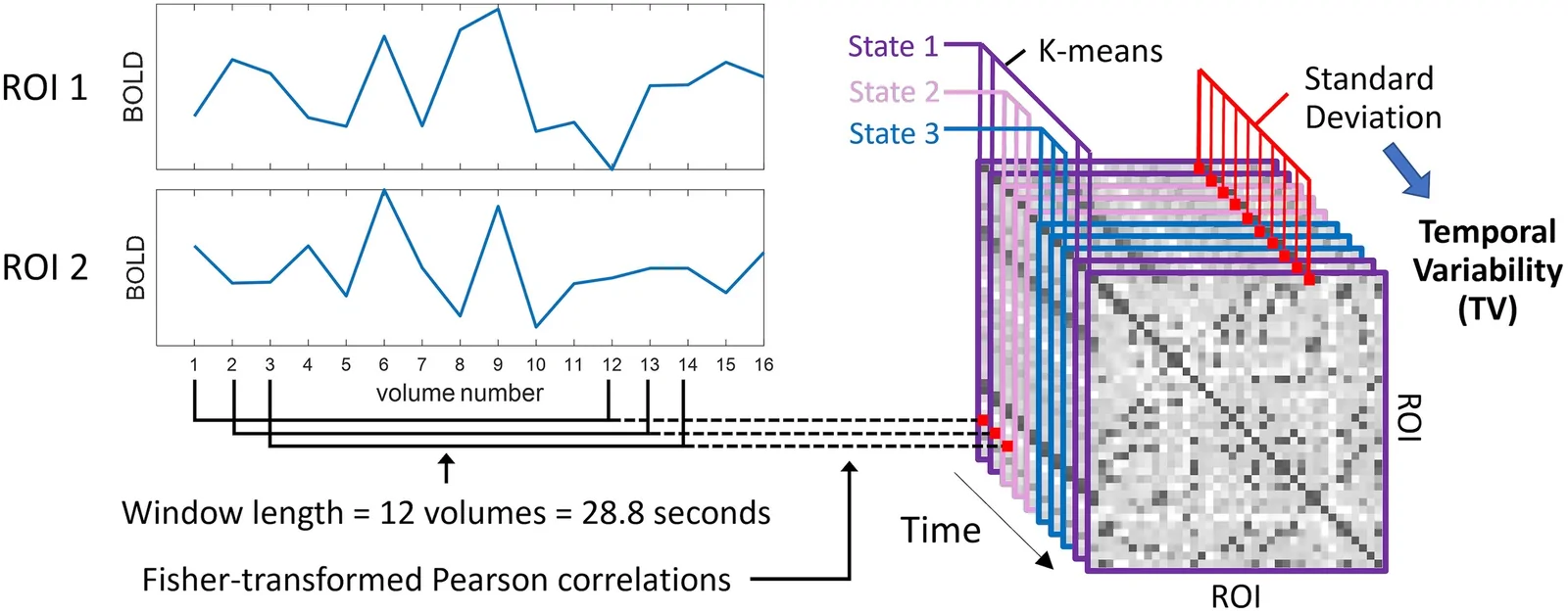
**Estimation of dFC.** Overview of methods for computing measures of dFC. dFC was estimated using a sliding window method where the dFC of a given ROI pair for overlapping time windows was computed as the Fisher-transformed Pearson correlation of their BOLD signals over the specified time window. BOLD, blood oxygen level dependent signal; ROI, region of interest.

dFC refers to time-varying inter-regional correlations in the fMRI BOLD signal. While several techniques exist for generating and analyzing dFC data from functional imaging, the most straightforward and commonly used method is the sliding-window approach, which was used in this study.^36,73,74^ An overview of the methods used to compute dFC measures is shown in Fig. 1. Three-dimensional N×N×T dFC matrices (where *N* is the number of nodes and *T* is number of time windows) were generated from mean BOLD time-series per ROI using a custom MATLAB script available at https://github.com/isaacfalconer/dfc_toolbox. The N×N matrix corresponding to each 30-s time window (actual window length = 12 timepoints = 28.8 s) was generated by computing the Pearson correlation between BOLD signals of each pair of nodes within the respective 30-s time window. 86 AAL3 parcels corresponding to all cortical ROIs were included in this study. Thus, with 164 time windows (175 functional volumes → 164 overlapping full 28.8 s windows), the sliding window procedure generated an 86 × 86 × 164 dFC matrix for each participant. Using these data, three analyses were performed: (ⅰ) TV, which measures the magnitude of fluctuations in dFC over time, (ⅱ) CS, which measures the mean duration of stable community structures, and (ⅲ) a dFC states analysis, which uses a clustering algorithm to identify transient recurring connectivity patterns.

### Temporal metrics

Using these dFC matrices, two temporal metrics of dFC were computed for each patient: TV and CS.

#### Temporal variability

**Figure 2:**
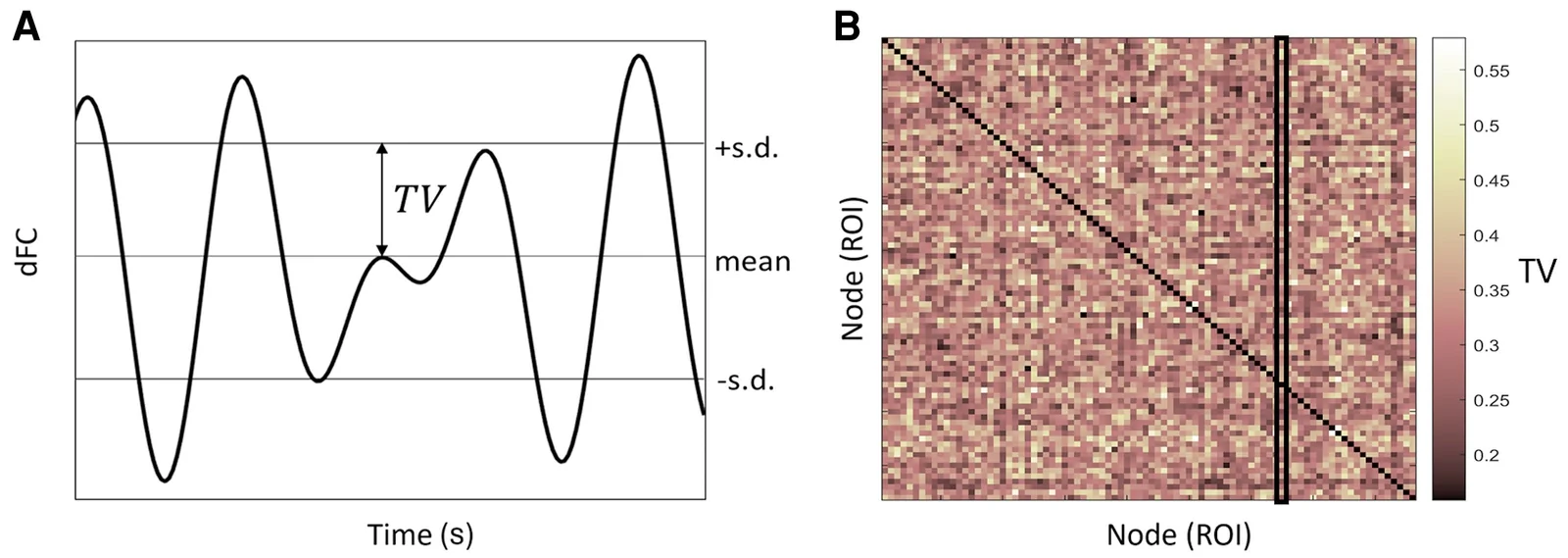
**Definition of TV.** (**A**) The TV for a functional connection (ROI-ROI) is defined as the SD of dFC over time. (**B**) Node TV is defined as the mean TV of all the ROI’s functional connections. Subject-level mean TV is computed as the mean of all node TV values (i.e. the mean of the entire matrix in panel (**B**). Each data point in the heatmap corresponds to one pair of nodes (*N* = 86) in a single representative subject. dFC, dynamic functional connectivity; ROI, region of interest.

TV is a measure of the magnitude of variation in region-to-region synchronization over all 30-s time windows (Fig. 2). For each node pair, a standard deviation (SD) of dFC over time was calculated, and subject-level mean TV was computed as the average of these standard deviations over all node pairs in the network. For each subject, heavily lesioned ROIs (i.e. > 50% damage) and all of their functional connections were excluded from the TV calculation. Proportion spared was calculated for each ROI as $(V_{\mathrm{tot}}-V_{L})/V_{\mathrm{tot}}$ and regions with proportion spared <0.5 were excluded.

#### Community stability

**Figure 3:**
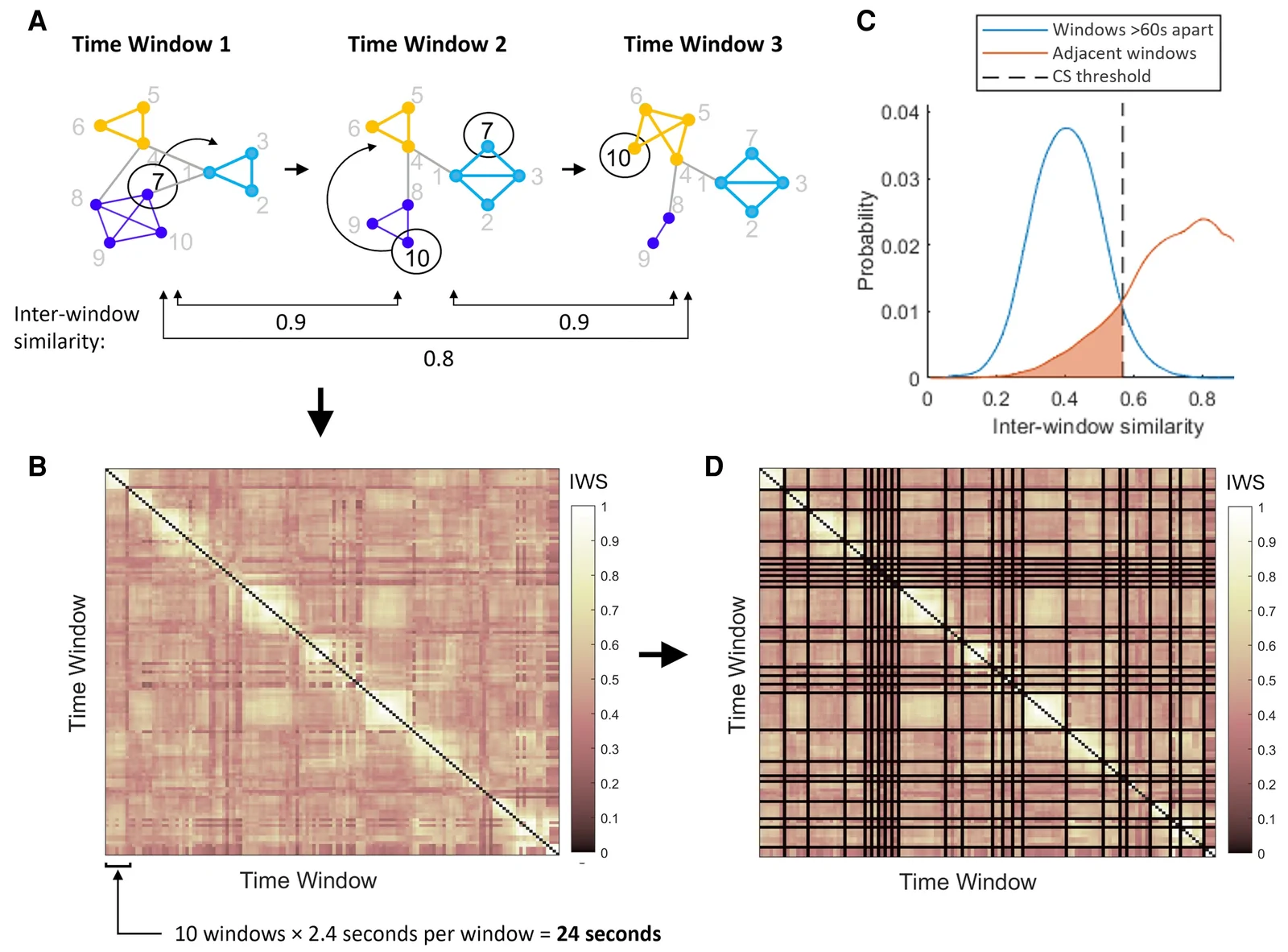
**Schema of CS analysis.** (**A**) The community structure for each time window was determined using the Louvain community detection algorithm. IWS was determined by the proportion of nodes with the same community membership between two windows. (**B**) IWS was computed for every pair of time windows. (**C**) Probability distributions of IWS for pairs of windows >60 s apart (blue curve) and adjacent windows (red curve) were used to define a CS threshold. (**D**) Points of low stability (black lines) were identified based on CS threshold. For panels (**B** and **D**), each data point in the heat map corresponds to a single time window (*N* = 164) for a single representative subject. IWS, inter-window similarity.

CS was determined based on changes over time in dFC community structure, which was determined using the Louvain community detection algorithm (Blondel *et al*.^82^). For computing CS, the inter-window similarity (IWS) between two-time windows was defined as the proportion of nodes belonging to the same communities in both time windows (Fig. 3A). For example, in Fig. 3A, the community structures of time windows 1 and 2 differ by a single node (node 7) out of a total of 10 nodes and thus have an IWS of 0.9. IWS was computed for all pairs of time windows, producing a T×T matrix (Fig. 3B). A CS threshold was defined based on the probability distribution of IWS for adjacent window pairs relative to that of distant window pairs. A pair of windows was considered distant if they were >60 s apart, which ensured that they were not correlated due to temporal proximity (see extent of red and orange regions along the diagonal in Fig. 3C). Points of low stability were defined by window pairs in close temporal proximity (≤2 windows apart) with below-threshold IWS (Fig. 3D). CS was computed as the mean length of time between these points of low stability (i.e. the mean duration of stable community structures). Only right hemisphere regions were included in the CS analysis for two reasons: (ⅰ) the community membership of lesioned ROIs is not meaningful and is likely to either remain unchanged or change erratically, both of which may bias the results, and (ⅱ) IWS is sensitive to the total number of nodes, and thus exclusion of varying numbers of lesioned regions (as was done for the TV analysis) may also bias results. Inclusion of only the right (unlesioned) hemisphere ensured that no lesioned tissue was included in the analysis and an equal number of regions was included for all subjects. In contrast, TV is much less sensitive to the number of regions included because it is simply a mean of standard deviations computed per node (as opposed to CS which is computed on a global network level), and thus excluding only lesioned regions was sufficient to prevent lesion effects while maximizing inclusion of relevant data.

As a clarifying point, TV and CS are neither mutually exclusive nor in opposition with one another, i.e. high TV does not imply low CS. TV measures the magnitude of dFC fluctuations and is not concerned with the frequency of those fluctuations. In contrast, CS measures the duration of stable community structures and is not necessarily concerned with the magnitude of difference between community structures.

### dFC states analysis

A third analysis investigated dFC states, i.e. recurring transient patterns of connectivity. This analysis differs from the CS analysis in that it is not concerned with the temporal ordering or temporal proximity of time windows, and instead groups time windows into states based on similarities in their connectivity patterns. These dFC states were identified using k-means clustering with the *kmeans* function from the Statistics and Machine Learning Toolbox in MATLAB.^75^ To identify the optimal k value, clustering was performed with k ranging from 1 to 25 on a concatenated dFC matrix (N×N×4920, where *N* is the number of nodes in the respective network) that included all time windows for all participants. A k of three was chosen by a combination of two methods: (ⅰ) the elbow plot method based on the within-cluster sum of squared distances and (ⅱ) visual inspection of clusters plotted as the first two principal components from a principal components analysis (PCA) performed on the dFC data using the *pca* function from the Statistics and Machine Learning Toolbox in MATLAB^75^ (Supplementary Fig. 1). This procedure was used previously for a different population of participants with PSA which also identified three dFC states.^76^ Input to the PCA consisted of the concatenated dFC matrix reshaped to a two-dimensional matrix with N^2^ columns and 4920 rows. To rule out effects of lesion size on the clustering procedure (e.g. clustering together of windows corresponding to patients with large lesions due to zero values in lesioned regions), the k-means algorithm was first run on the right (non-lesioned) hemisphere dFC only, producing a vector of cluster (i.e. state) labels per window. Using these state label assignments, subsequent analyses of mean dFC per state were performed on the full bilateral dFC. Fractional occupancy (FO) per subject was calculated for each state as the number of time windows clustered into the respective state divided by the total number of windows (164), consistent with previous dFC studies.^77,78^

After identifying dFC states and computing their FO, each state was characterized based on mean dFC and the graph metric, modularity. Modularity measures the strength of within-module (i.e. subgroup of nodes) connectivity relative to between-module connectivity strength. Additionally, for each state, a mean connectivity matrix was computed as the mean dFC all time windows corresponding to that state. Connectivity of 34 regions corresponding to an extended language network (see^25^ for details) was extracted from these mean dFC matrices for visualization. Due to the large number of connections (3655) included in this study, language network regions, which are particularly relevant to PSA, were visualized alone to maximize interpretability. However, full connectivity maps per state are shown in Supplementary Fig. 2.

### Statistical analyses

Relationships of dFC measures with baseline naming accuracy and aphasia severity were evaluated using simple linear regression models. Linear mixed effects models were used to evaluate the relationship between baseline dFC measures and treatment response. This included five separate models, each with one of the following variables as the predictor of interest: TV, CS, State 1 FO, State 2 FO and State 3 FO. Each model included naming accuracy as the response variable and fixed effects of session (i.e. time), aphasia severity, lesion volume and an interaction of session with each of the covariates. Aphasia severity and lesion volume were accounted for in these models as they are known predictors of treatment response. A random effect of participant was included to account for random variation in baseline naming performance. To avoid overly complex models, static functional connectivity (sFC) and structural connectivity measures were not included in the main models. However, a series of models with TV as the predictor of interest were run including covariates of white matter tract fractional anisotropy values and sFC of functional networks to evaluate for these measures accounting for some of the effect of TV (Supplementary Tables 8 and 9). A significant interaction between session and the respective dFC measure (i.e. changes in the rate of improvement in naming performance as a function of the dFC measure) was interpreted as a significant effect of the dFC measure on treatment response. For characterization of dFC states, mean dFC and modularity were compared between states using two-sample *t*-tests. A subject-level mean for each state (i.e. averaged over all time windows corresponding to the respective state for each subject) was used for these comparisons.

## Results

### Sample characteristics

Table 1 provides an overview of the participants’ demographic and clinical characteristics. The mean age was 61.47 years (SD = 10.74), with an average time since stroke of 52.8 months (SD = 50.13). Most participants were male (67%) and right-handed (93%). Aphasia severity at baseline, as indexed by the Western Aphasia Battery-Revised Aphasia Quotient (WAB-R AQ), varied widely across participants, with scores ranging from 11.7 to 95.2 (mean = 59.84, SD = 24.97). Changes in naming accuracy after treatment ranged from −6% to 55% improvement, with an average increase of 25% (SD = 17%) across all participants.

**Table 1:** Participant demographic information

Subject	Age	MPO	Education (years)	Gender	Handedness	WAB AQ	Change in naming accuracy	Lesion Volume (cm^3^)	Number of Sessions
P01	55	12	16	M	R	87.2	55%	62.7	17
P02	50	29	16	F	L	25.2	0%	286.7	18
P03	63	62	16	F	R	52	31%	197.7	18
P04	79	13	16	M	R	74.1	50%	91.3	13
P05	67	8	18	M	R	30.8	−6%	172	18
P06	49	113	16	M	R	66.6	34%	319.9	14
P07	55	137	16	M	R	48	19%	222.3	18
P08	49	52	12	M	R	82.8	28%	100.2	9
P09	71	37	16	F	R	95.2	46%	14.1	18
P10	53	12	16	F	R	80.4	37%	81.5	13
P11	78	22	18	M	R	92.1	19%	36.6	18
P12	68	104	12	M	R	40	7%	216.2	14
P13	42	18	13.5	M	L	92.7	52%	14.4	18
P14	64	24	13	F	R	64.4	50%	123.8	14
P15	71	74	12	F	R	87.2	16%	184.2	17
P16	49	70	12	M	R	33.6	7%	342.2	18
P17	61	152	16	M	R	74.3	48%	184.1	11
P18	70	152	16	F	R	78	38%	84.9	18
P19	80	22	18	M	R	28.9	17%	112.8	18
P20	48	14	16	F	R	13	42%	186.9	18
P21	65	16	18	M	R	11.7	21%	272.4	18
P22	62	12	16	M	R	65.4	10%	105.8	17
P23	60	24	16	M	R	45.2	4%	186	17
P24	69	170	16	M	R	40.4	13%	228.4	18
P25	76	33	18	F	R	37.5	6%	192.8	18
P26	64	115	12	F	R	58	14%	136.6	18
P27	65	17	12	M	R	84.3	29%	37.5	18
P28	62	15	12	M	R	56	41%	80.1	18
P29	40	26	16	M	R	90.1	9%	269.5	18
P30	59	29	14	M	R	60	24%	202.7	18
**Mean**	**61.47**	**52.8**	**15.15**	**20 M**	**28 R**	**59**.**84**	**25%**	**158**.**2**	**16.6**
**SD**	**10.74**	**50.13**	**2.11**	**10 F**	**2 L**	**24**.**97**	**17%**	**89**.**8**	**2.44**

Bold values correspond to group-level mean and SD for the cohort of 30 participants.

Abbreviations: Change in accuracy %, change in accuracy on the treatment probes measured as a percentage (i.e. post-treatment accuracy percentage minus pre-treatment accuracy percentage); F, female; L, left; M, male; MPO, Months post-stroke onset; R, right; SD, standard deviation; WAB-R AQ, Western Aphasia Battery-Revised Aphasia Quotient.

### Temporal metrics of dFC

**Figure 4:**
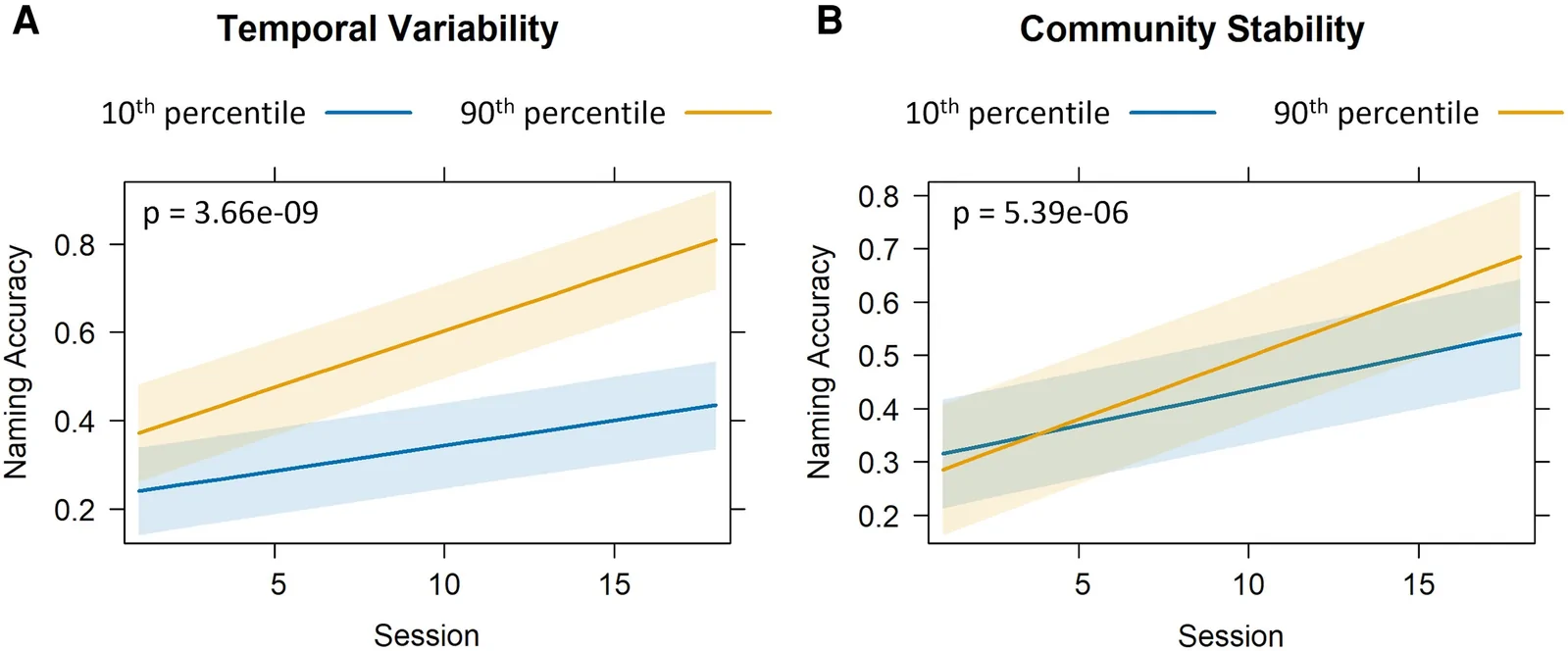
**Temporal metrics as predictors of naming accuracy gains.** Linear mixed effects model-predicted naming accuracy showing interaction effects of dFC temporal metrics with session (*N* = 30 subjects). (**A**) Model-predicted naming accuracy as a function of session. The blue line represents model predictions for an individual with TV in the 10th percentile and the gold line represents model predictions for and individual with TV in the 90th percentiles. Blue and gold lines do not correspond to subgroups or specific subjects, but rather to the model-predicted naming accuracy corresponding to the specified state 2 FO level. (**B**) Predicted naming accuracy as a function of session for patients with CS in the 10th (blue line) and 90th (gold line) percentiles. Shaded regions represent 95% confidence intervals.

TV, but not CS, was significantly associated with baseline naming accuracy with higher TV participants having higher baseline naming accuracy (Supplementary Table 1). Neither measure had a significant relationship with aphasia severity (Supplementary Table 2). Linear mixed effects models revealed significant associations between the dFC measures, TV and CS, and improvements in naming accuracy over the course of treatment. A summary of all model results, including temporal metrics and states analysis, is shown in Table 2. Full model results for TV and CS are shown in Supplementary Tables 3 and 4, respectively. Fixed effects in the two models explained 69% and 63% of the variance in naming accuracy, respectively. First, session was significantly associated with naming accuracy indicating that, overall, patients showed an increase in naming accuracy over time, consistent with the previously reported findings from the same dataset.^59^ Significant interactions with session for both TV and CS indicated that greater TV and greater CS were both associated with a greater rate of improvement in naming accuracy (Fig. 4). Consistent with previous studies using the same dataset,^9,25^ aphasia severity and lesion volume were also significantly associated with naming accuracy improvement in both models, with higher WAB AQ (i.e. less severe aphasia) and smaller lesions predicting greater rates of improvement. Overall, these results show that patients with (ⅰ) a greater magnitude of variation in region-to-region synchronization (i.e. higher TV) and (ⅱ) a tendency to maintain a given dFC community structure for greater lengths of time (i.e. higher CS) showed greater response to aphasia therapy.

**Table 2:** Summary of model results for linear mixed effects models with dFC temporal metrics and state fractional occupancies as predictors of interest

	Predictor	Beta	*P*-value	Partial *η*^2^
Model 1	Temporal Variability	0.451	3.66e−09***	0.0722
Model 2	Community Stability	0.00459	5.39e−06***	0.0436
Model 3	State 1 Fractional Occupancy	−1.87e−05	0.271	0.00261
Model 4	State 2 Fractional Occupancy	7.89e−05	6.23e−04***	0.0249
Model 5	State 3 Fractional Occupancy	−2.76e−05	0.133	0.00486

Significance levels: <0.001***.

Each row corresponds to a separate mixed effects model and the statistics shown are for interaction effects between the predictor of interest and session, indicating an effect on the rate of improvement in naming performance. Partial *η*^2^ is an estimate of the effect size for each predictor in its respective model.

### dFC states analysis

**Figure 5:**
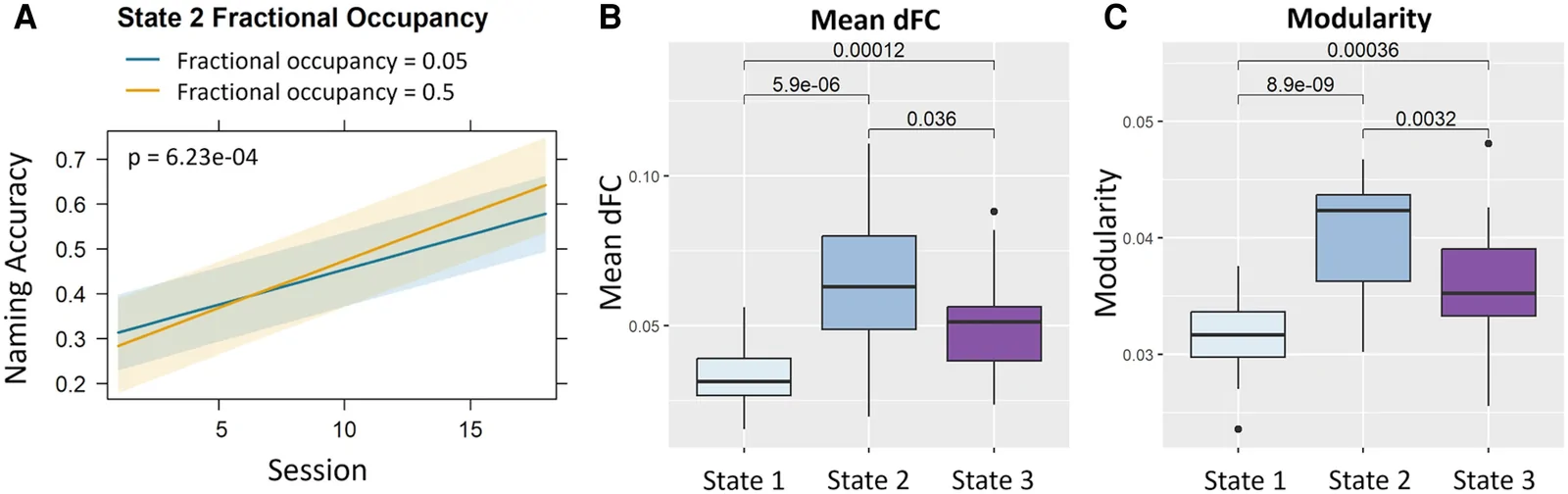
**dFC state 2 analyses.** (**A**) Naming accuracy predictions by a linear mixed effects model as a function of session (*N* = 30). The blue line indicates predicted accuracy for a patient with a state 2 FO of 0.05 and the gold line indicates predicted accuracy for a subject with a state 2 FO of 0.5. Blue and gold lines do not correspond to subgroups or specific subjects, but rather to the model-predicted naming accuracy corresponding to the specified state 2 FO level. (**B**) Comparison of mean dFC for each of the three states (Welch’s two sample *t*-tests: state 1 and state 2: *P* = 5.9e−06, *t* = −7.43, state 1 and state 3: *P* = 1.2e−04, *t* = −3.71, state 2 and state 3: *P* = 0.036, *t* = 3.06). (**C**) Comparison of modularity of time windows corresponding to each of the three states (Welch two sample *t*-tests: state 1 and state 2: *P* = 8.9e−09, *t* = −5.57, state 1 and state 3: *P* = 3.6e−04, *t* = −4.28, state 2 and state 3: *P* = 3.2e−03, *t* = 2.17). For panels (**B** and **C**), subject-level means of mean dFC or modularity were compared between states. Because some subjects did not occupy all three states, the number of observations in each statistical test varied by state (state 1: *N* = 28, state 2: *N* = 23, state 3: *N* = 25). dFC, dynamic functional connectivity.

**Figure 6:**
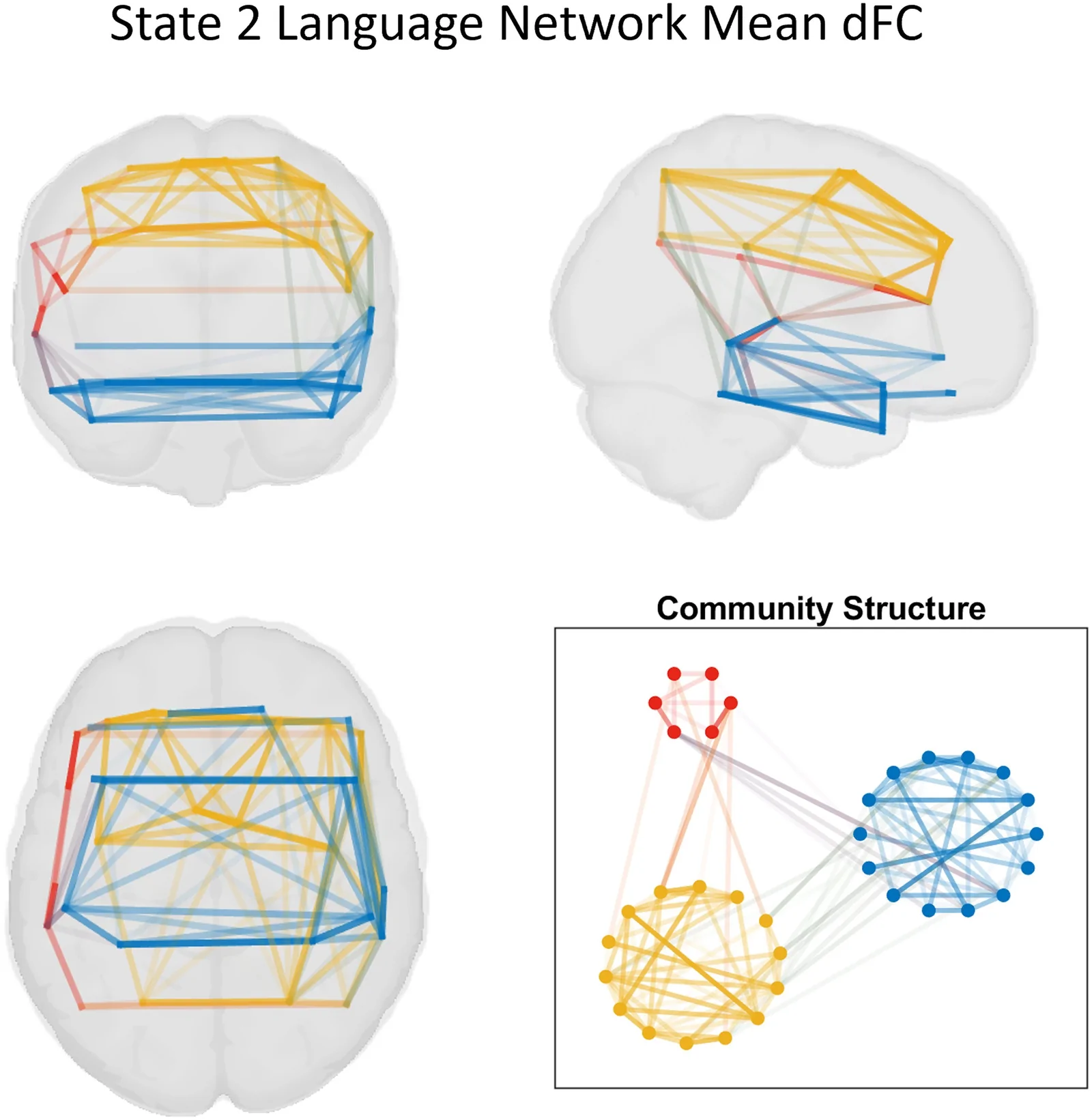
**State 2 language network.** Mean dFC of an extended language network extracted from state 2 dFC windows. See Supplementary Fig. 2 for whole cortex mean dFC of all three states.

Three dFC states were identified and the FO of (i.e. time spent in) these states was computed for each subject. No significant associations between either aphasia severity or baseline naming accuracy and FO of the three dFC states were found (Supplementary Tables 1 and 2). Model results for the predictors of interest (i.e. state FO) are shown in Table 2 and full model results are shown in Supplementary Tables 5–7. A significant interaction between session and state 2 FO was found, indicating that time spent in this state predicted better treatment response (Fig. 5A). No significant interactions were observed for FO of states 1 and 3. State 2 had significantly greater mean dFC and modularity compared with both State 1 and State 3 (Fig. 5B, C). State 2 mean dFC in the language network is shown in Fig. 6 (see Supplementary Figs 2 and 3 for mean dFC of all states). It is a densely connected state with two highly segregated communities (spatially separated into dorsal and ventral regions) and a third left-hemisphere community situated as a hub between them. This third community was unique to State 2 and consisted of peri-sylvian areas that included core language regions (inferior frontal gyrus and temporal and parietal regions centred on the temporoparietal junction). State 2 dFC was also notable for relatively weak interhemispheric connectivity between peri-sylvian regions.

## Discussion

This study investigated temporal metrics of dFC in patients with PSA and their relationship to rates of improvement in naming accuracy over the course of an aphasia treatment aimed at improving word-finding. We found that both higher TV and greater CS at baseline were associated with greater rates of improvement in naming accuracy. Additionally, a state-based dFC analysis revealed that the time spent in a state characterized by higher overall connectivity and modularity was related to better treatment response. These findings suggest that baseline dFC metrics may reflect an individual’s capacity for functional network reorganization, illustrating their potential as biomarkers for predicting therapy outcomes. As reported in greater detail in a previous publication, significant improvements in naming accuracy were observed with the treatment.^59^

### Temporal metrics: temporal variability and community stability

Higher TV, which reflects greater short-term flexibility in functional network dynamics, was significantly associated with better treatment response. This finding is consistent with prior work linking dFC flexibility to recovery in stroke,^54^ motor learning,^55^ and cognitive function in other neurological disorders.^26-33^ For instance, studies in stroke recovery have shown that lower dFC flexibility correlates with persistent deficits in motor and cognitive domains. Similarly, reduced dFC flexibility has been linked to cognitive deficits in Alzheimer’s disease, schizophrenia, and major depressive disorder, where lower network variability is associated with poorer outcomes.^54^ In PSA, higher TV may indicate a neural system capable of reorganizing and forming new functional connections, enhancing recovery potential, a proposal that the remainder of this section will outline.

**Figure 7:**
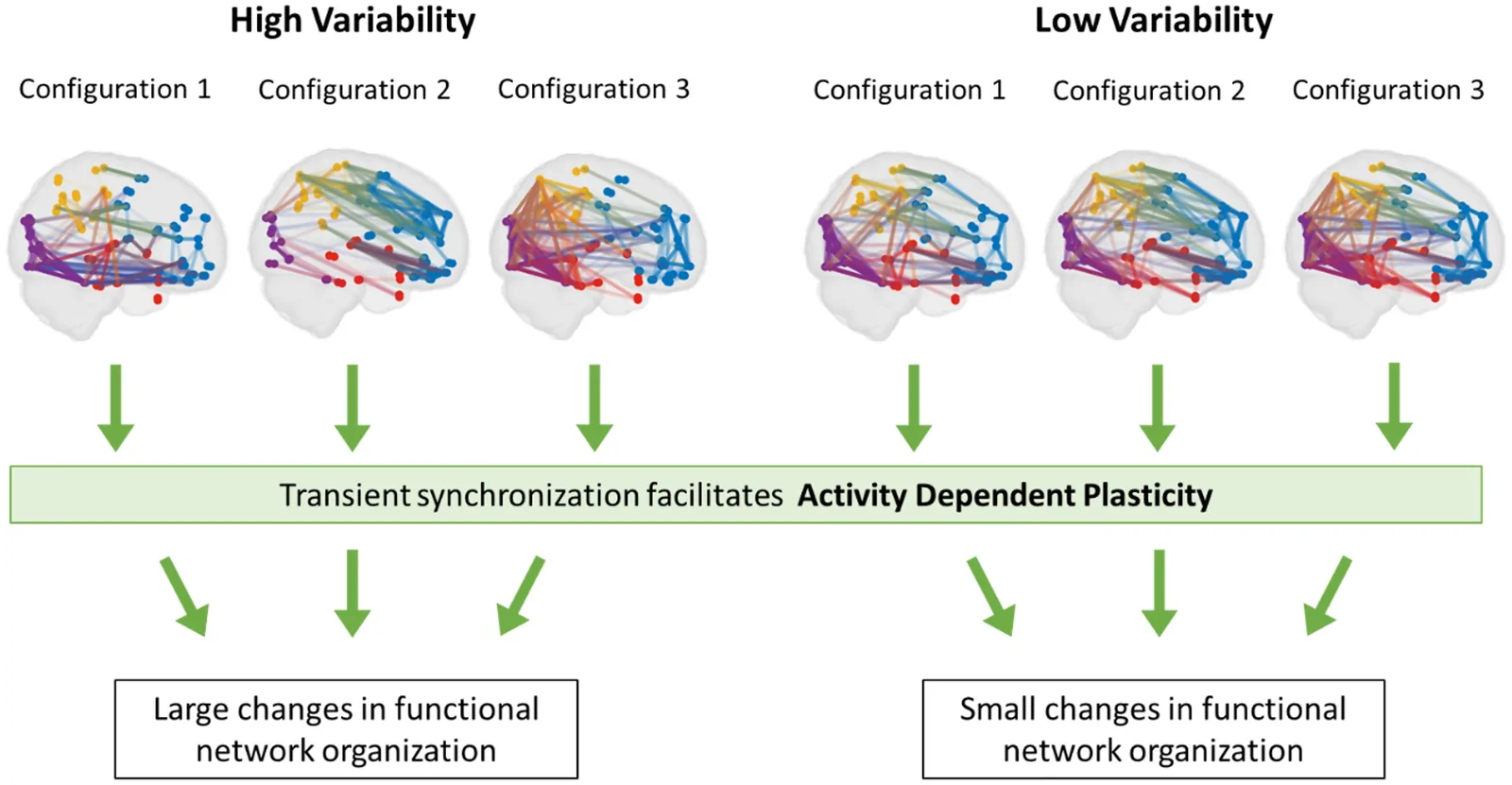
**Proposed mechanism of the relationship between dFC TV and functional network plasticity.** Higher TV (left) provides greater diversity in the patterns of inter-regional synchronization, each of which provide a unique opportunity for synaptic plasticity in the connections between synchronized regions. These transient opportunities for facilitated plasticity accumulate over time leading to greater functional network changes supporting recovery, in contrast with low TV (right) which provides a smaller variety of opportunities for plasticity and less accumulated change over time. dFC, dynamic functional connectivity.

Considering our findings together with previous studies, we propose the following mechanism for the link between TV and behavioural improvements post-stroke: First, transient synchronization between regions facilitates modulation of their long-term connectivity, driven by activity and/or stimulation (e.g. therapy, practice, exposure to specific stimuli, etc.). This aligns with activity-dependent plasticity principles, where neural co-activation influences synaptic strengthening or weakening over time.^79,80^ A relevant macroscale parallel comes from Boutin *et al*.,^81^ who found that stronger transient synchronizations between key regions during sleep were associated with improved motor sequence learning the following day. Thus, it is not a great leap to suppose that macroscale transient synchronizations between regions may facilitate activity-dependent plasticity between them. Second, given the facilitation of activity-dependent plasticity by transient synchronizations, a greater diversity of connectivity configurations (i.e. higher TV) provides a wider range of opportunities for facilitation of functional network plasticity (i.e. long-term change of functional networks). A schema of this proposed mechanism shown in Fig. 7 illustrate how higher TV may facilitate plasticity supporting treatment-induced recovery, i.e. improved naming performance in this study.

In addition to TV, greater CS at baseline, reflecting longer-lasting, stable network configurations, was also associated with greater improvement in naming accuracy. The result suggests that PWA who tends to maintain a given spatial configuration of dFC (i.e. community structure) for longer lengths of time are able to benefit more from aphasia therapy. This is consistent with previous studies outside the context of PSA that have shown that greater network stability is associated with better learning and overall cognitive ability.^42,43,45^ Because the analyses reported here only include resting-state data it is difficult to infer specific relationships with behaviour. However, it is possible that greater CS reflects enhanced attentional control, which may help patients better engage in behavioural treatment activities. Interestingly, a post-hoc analysis revealed that CS was highly correlated with sFC of both the dorsal attention and salience networks, providing additional evidence for this interpretation (Supplementary Fig. 4). Additionally, within the framework described above for TV, greater network stability reflects inter-regional synchronizations that persist for longer periods of time, which may prolong any influence on plasticity mechanisms (i.e. long-term potentiation and long-term depression). Thus, when coupled with high TV, it is possible that higher CS also facilitates network changes underlying recovery. Overall, this result was consistent with our prediction based on the hypothesis that CS reflects greater network stability supporting productive engagement in treatment activities, which may be mediated by greater attentional control.

It is important to note that the temporal resolution of dFC measures does not capture the millisecond-scale dynamics of real-time language processing. Instead, these metrics reflect the broader capacity of the brain’s functional networks to dynamically transition between different states over time. This dynamic flexibility is critical for long-term network reorganization, which ultimately supports functional recovery. Rather than measuring discrete language processing steps, dFC metrics provide insights into the underlying neural plasticity mechanisms that enable treatment-induced improvements.

### dFC states analysis

In addition to the dFC temporal metrics, a k-means clustering analysis was performed to identify recurring transient dFC configurations. Of the three states identified, greater FO of (i.e. time spent in) state 2 was found to relate to better treatment response. This state was characterized by higher mean dFC and higher modularity compared with states 1 and 3. The latter finding is consistent with previous findings linking greater network modularity to better recovery outcomes.^46,48,57^ It also expands on our previous observation that patients with PSA spend more time in a less modular state compared with healthy controls.^76^ These results support the hypothesis that a preference for highly modular states reflects better function and supports treatment response. Further characterization of the three states by extracting mean dFC for an extended language network revealed a distinct community of left hemisphere peri-sylvian areas that included core language regions and was present in only state 2. This community was situated as a hub between two highly segregated communities composed of dorsal and ventral regions, respectively. Given that state 2 had higher overall dFC, this language network configuration, with a community of core language regions distinct from more peripheral ones, suggests that preferentially increased dFC within this core language network, as well as within resting state networks in general, supports treatment-induced recovery. State 1 was characterized by overall weaker connectivity relative to states 2 and 3 and was less segregated into well-defined communities. State 3 was characterized by greater interhemispheric connectivity relative to states 1 and 2. Neither FO of state 1 or FO of state 3 were significantly associated with treatment response.

### Clinical implications

These findings suggest that baseline dFC properties could help identify patients who are more likely to benefit from intensive language therapy. Individuals with higher TV may have greater neural flexibility, making them more responsive to targeted interventions whereas those with lower TV may require additional strategies to enhance network reorganization. Similarly, greater CS may indicate stronger attentional control, suggesting that patients with lower CS might benefit from interventions that enhance cognitive engagement during therapy. Future work should explore how dFC metrics can be integrated into clinical decision-making to optimize aphasia rehabilitation strategies.

### Limitations and future directions

Several important limitations should be considered in the interpretation of the findings reported here. First, the use of resting-state functional MRI data in dFC analyses means that patterns and temporal properties of dFC cannot be directly related to any specific cognitive or behavioural factors. While our interpretation of the TV finding does not necessarily depend on such a relationship, we have speculated that our CS result may be related to cognitive factors such as attentional control. Future investigations of task- and/or naturalistic stimuli-based dFC could clarify any relationships between relevant cognitive factors and CS. A second limitation of this study is that it did not include healthy controls for comparison. A separate study is currently investigating TV and CS in individuals with PSA compared with healthy controls to evaluate if stroke itself or any compensatory or adaptive mechanisms result in alterations of the dFC temporal metrics used in the study. A third limitation is that this study only examined temporal metrics of global network connectivity. Future studies investigating these measures on an individual region level may reveal spatial variation in temporal dynamics and may identify specific connections whose TV is particularly important for language recovery. Additionally, more studies on the global level examining TV of graph metrics may also yield insights into how connectivity patterns of functional networks fluctuate and how these fluctuations relate to recovery.

## Conclusion

Overall, our results demonstrate the relevance of temporal properties of dFC to treatment-induced PSA recovery. They suggest that patients who have a greater magnitude of variation in region-to-region synchronization over time, but whose dFC community structure remains stable for greater lengths of time, benefit more from treatment than those with a smaller magnitude variation but more frequent changes in community structure. Taking this result together with a similar result in the context of post-stroke motor recovery^54^ we have proposed the following mechanism for the relationship between TV and treatment response: (ⅰ) Transient synchronizations between brain regions facilitate activity-dependent plasticity and (ⅱ) greater diversity in transient synchronizations over time presents a wider range of opportunities for activity-dependent plasticity, thus facilitating long-term functional network plasticity. With respect to the TV and dFC states analysis, the results of this study are generally consistent with those of our previous study comparing a different sample of patients to healthy controls, further highlighting TV and a preference for higher modularity dFC states, important factors relevant to brain function and recovery in PSA.

## Supplementary Material

fcag279_Supplementary_Data

## Data Availability

Deidentified raw imaging data, lesion information and behavioural data used in this study are available on the Open Science Framework (OSF): https://osf.io/wcuj8. Software used for image preprocessing is freely available at https://fmriprep.org/en/1.4.1/and https://github.com/neurolabusc/Clinical. Software used for additional preprocessing and generation of mean BOLD timeseries per ROI is freely available at https://web.conn-toolbox.org/. Custom MATLAB code used for estimation of dFC and subsequent analyses is available at https://github.com/isaacfalconer/dfc_toolbox. The Brain Connectivity Toolbox, which was used to compute graph measures, is freely available at http://mathworks.com/matlabcentral/fileexchange/61173-brain-connectivity-toolbox. Statistical analysis code is available at https://osf.io/zja2b/. Legal copyright restrictions prevent public archiving of the Western Aphasia Battery—Revised which can be obtained from the copyright holders in the cited references (Kertesz, 2007).
