# Supplementary material for: Network flexibility facilitates treatment-induced recovery in post-stroke aphasia
Source: Brain Commun. 2026 Sep 18;8(5):fcag279. doi: 10.1093/braincomms/fcag279 (PMC13586764; doi:10.1093/braincomms/fcag279)
Supplement: Supplementary file 1 [file fcag279_supplementary_data.pdf]

Supplementary Material

Supplementary Table 1. Linear regression models with dFC measures as predictors of baseline naming accuracy.

	Estimate	Std. Error	t value	Pr(>|t|)
**Temporal variability**				
(Intercept)	7.35e-16	0.167	4.39e-15	1
TV	0.435	0.17	2.55	0.0164
**Community stability**				
(Intercept)	-9.87e-17	0.18	-5.47e-16	1
CS	0.239	0.184	1.3	0.203
**State 1 fractional occupancy**				
(Intercept)	-3.26e-17	0.185	-1.76e-16	1
State 1 FO	-0.0653	0.189	-0.346	0.732
**State 2 fractional occupancy**				
(Intercept)	-5.05e-17	0.185	-2.73e-16	1
State 2 FO	-0.109	0.188	-0.58	0.567
**State 3 fractional occupancy**				
(Intercept)	-2.79e-17	0.184	-1.52e-16	1
State 3 FO	0.156	0.187	0.834	0.411

Supplementary Table 2. Linear regression models with dFC measures as predictors of aphasia severity (WAB AQ).

	Estimate	Std. Error	t value	Pr(>|t|)
**Temporal variability**				
(Intercept)	4.73e-16	0.179	2.64e-15	1
TV	0.265	0.182	1.45	0.157
**Community stability**				
(Intercept)	-7.03e-17	0.178	-3.95e-16	1
CS	0.289	0.181	1.6	0.122
**State 1 fractional occupancy**				
(Intercept)	2.48e-17	0.182	1.36e-16	1

	Estimate	Std. Error	t value	Pr(>|t|)
State 1 FO	-0.204	0.185	-1.1	0.279
**State 2 fractional occupancy**				
(Intercept)	-2.9e-19	0.186	-1.56e-18	1
State 2 FO	-0.00318	0.189	-0.0168	0.987
**State 3 fractional occupancy**				
(Intercept)	1.84e-17	0.181	1.01e-16	1
State 3 FO	0.227	0.184	1.23	0.229

Supplementary Table 3. Full linear mixed effects model results for temporal variability model (model formula: accuracy ~ Session*TV + Session*wab_aq + Session*lesion_volume + (1|ID))

	**Estimate**	**Std. Error**	**df**	**t value**	**Pr(>|t|)**
(Intercept)	-1.52	0.923	29.1	-1.65	0.111
Session	-0.135	0.0255	465	-5.31	1.71e-07
TV	3.73	2.74	29.1	1.36	0.184
wab_aq	0.00809	0.00166	28.9	4.88	3.59e-05
lesion_volume	2.4e-06	3.58e-06	29	0.669	0.509
Session:TV	0.451	0.075	465	6.01	3.66e-09
Session:wab_aq	1.14e-04	4.3e-05	464	2.65	0.00834
Session:lesion_volume	-4.14e-07	9.55e-08	464	-4.33	1.8e-05

Supplementary Table 4. Full linear mixed effects model results for community stability model (model formula: accuracy ~ Session*comm_stability + Session*wab_aq + Session*lesion_volume + (1|ID))

	**Estimate**	**Std. Error**	**df**	**t value**	**Pr(>|t|)**
(Intercept)	-0.186	0.29	28.4	-0.64	0.527
Session	-0.00987	0.00695	464	-1.42	0.156
comm_stability	-0.0179	0.0402	28.5	-0.445	0.66
wab_aq	0.00888	0.00183	28.4	4.86	3.95e-05
lesion_volume	2.71e-06	4.07e-06	28.4	0.666	0.511
Session:comm_stability	0.00459	9.97e-04	465	4.6	5.39e-06
Session:wab_aq	1.29e-04	4.35e-05	464	2.98	0.00306
Session:lesion_volume	-3.64e-07	9.74e-08	464	-3.74	2.1e-04

Supplementary Table 5. Full linear mixed effects model results for State 1 fractional occupancy model (model formula: accuracy ~ Session*state_1_FO + Session*wab_aq + Session*lesion_volume + (1|ID))

	**Estimate**	**Std. Error**	**df**	**t value**	**Pr(>|t|)**
(Intercept)	-0.292	0.172	28.5	-1.69	0.102
Session	0.0165	0.00416	464	3.96	8.77e-05
state_1_FO	5.45e-04	7.17e-04	28.4	0.761	0.453
wab_aq	0.00861	0.00182	28.5	4.73	5.57e-05
lesion_volume	1.37e-06	4.48e-06	28.5	0.307	0.761
<u>Session:state_1_FO</u>	-1.87e-05	1.7e-05	464	-1.1	0.271
<u>Session:wab_aq</u>	1.66e-04	4.39e-05	464	3.77	1.84e-04
<u>Session:lesion_volume</u>	-3.61e-07	1.07e-07	464	-3.38	8e-04

Supplementary Table 6. Full linear mixed effects model results for State 2 fractional occupancy model (model formula: accuracy ~ Session*state_2_FO + Session*wab_aq + Session*lesion_volume + (1|ID))

	Estimate	Std. Error	df	t value	Pr(>|t|)
(Intercept)	-0.261	0.182	28.4	-1.43	0.163
Session	0.0123	0.00425	464	2.89	0.00406
state_2_FO	-5.04e-04	9.14e-04	28.5	-0.551	0.586
wab_aq	0.00869	0.00183	28.4	4.76	5.21e-05
lesion_volume	2.61e-06	4.1e-06	28.4	0.637	0.529
<u>Session:state_2_FO</u>	7.89e-05	2.29e-05	465	3.45	6.23e-04
<u>Session:wab_aq</u>	1.7e-04	4.34e-05	464	3.92	1.02e-04
<u>Session:lesion_volume</u>	-3.75e-07	9.83e-08	464	-3.81	1.55e-04

Supplementary Table 7. Full linear mixed effects model results for State 3 fractional occupancy model (model formula: accuracy ~ Session*state_3_FO + Session*wab_aq + Session*lesion_volume + (1|ID))

	Estimate	Std. Error	df	t value	Pr(>|t|)
(Intercept)	-0.258	0.186	28.5	-1.39	0.175
Session	0.0187	0.00449	464	4.17	3.71e-05
state_3_FO	-2.98e-04	7.57e-04	28.5	-0.394	0.697
wab_aq	0.00874	0.0018	28.5	4.85	4.02e-05
lesion_volume	2.23e-06	4.29e-06	28.5	0.519	0.608

	Estimate	Std. Error	df	t value	Pr(>|t|)
Session:state_3_FO	-2.76e-05	1.83e-05	464	-1.51	0.133
Session:wab_aq	1.59e-04	4.38e-05	464	3.63	3.1e-04
Session:lesion_volume	-4.61e-07	1.05e-07	464	-4.37	1.53e-05

Supplementary Table 8. Linear mixed effects models including an interaction of session and fractional anisotropy of white matter tracts as predictors of naming accuracy. A separate model was run for each of 12 white matter tracts as well as one model for the mean fractional anisotropy of all 12 white matter tracts. With the exception of the left arcuate fasciculus model, higher TV remains significantly associated with a greater rate of improvement in naming accuracy in all models. For the left arcuate fasciculus, 22 patients did not have FA values due to their lesion location and size and were thus not included in the respective model resulting in very low statistical power for this model.

	Estimate	Std. Error	df	t value	Pr(>|t|)
**Corpus callosum forceps major**					
(Intercept)	-0.43	1.31	21.3	-0.327	0.746
Session	-0.117	0.0365	366	-3.2	0.00152
TV	0.978	3.68	21.3	0.266	0.793
fa_avg_ccmaj	-0.116	0.53	21.3	-0.22	0.828
wab_aq	0.0078	0.00179	21.2	4.35	2.74e-04
lesion_volume	-2.71e-07	4.37e-06	21.2	-0.062	0.951
Session:TV	0.365	0.104	367	3.51	5.03e-04
Session: fa_avg_ccmaj	0.0255	0.0147	367	1.74	0.0832
Session:wab_aq	8.77e-05	4.72e-05	366	1.86	0.0639
Session:lesion_volume	-5.17e-07	1.14e-07	366	-4.54	7.51e-06
**Corpus callosum foceps minor**					
(Intercept)	-1.74	0.943	28.1	-1.84	0.0757
Session	-0.145	0.0262	464	-5.54	5e-08
TV	3.49	2.72	28.1	1.28	0.209
fa_avg_ccmin	0.685	0.736	28	0.93	0.36
wab_aq	0.00766	0.00171	27.9	4.48	1.14e-04
lesion_volume	2.16e-06	3.56e-06	28	0.607	0.548
Session:TV	0.441	0.0752	464	5.86	8.69e-09
Session: fa_avg_ccmin	0.0318	0.0201	464	1.58	0.115
Session:wab_aq	9.36e-05	4.47e-05	463	2.09	0.037
Session:lesion_volume	-4.39e-07	9.66e-08	464	-4.55	6.85e-06

	Estimate	Std. Error	df	t value	Pr(>|t|)
**Left inferior fronto-occipital fasciculus**					
(Intercept)	-2.21	1.39	6.69	-1.59	0.157
Session	-0.194	0.038	166	-5.09	9.55e-07
TV	4.81	3.77	6.7	1.27	0.245
fa_avg_lifof	1.79	1.07	6.64	1.67	0.142
wab_aq	0.00363	0.00354	6.61	1.03	0.341
lesion_volume	-2.04e-06	8.67e-06	6.62	-0.236	0.821
Session:TV	0.656	0.104	166	6.3	2.55e-09
Session: fa_avg_lifof	0.0106	0.027	166	0.39	0.697
Session:wab_aq	-4.69e-05	8.33e-05	166	-0.563	0.575
Session:lesion_volume	-3.9e-07	2.08e-07	166	-1.88	0.0618
**Left inferior longitudinal fasciculus**					
(Intercept)	-1.66	1.31	22.3	-1.27	0.219
Session	-0.161	0.0356	383	-4.52	8.22e-06
TV	3.94	3.51	22.2	1.12	0.273
fa_avg_lilf	0.356	0.802	22.3	0.444	0.662
wab_aq	0.00732	0.0025	22.1	2.94	0.00763
lesion_volume	2.51e-06	6.47e-06	22.2	0.387	0.702
Session:TV	0.496	0.0942	382	5.26	2.41e-07
Session: fa_avg_lilf	0.0465	0.0218	382	2.13	0.0337
Session:wab_aq	4.22e-05	6.36e-05	382	0.662	0.508
Session:lesion_volume	-4.12e-07	1.73e-07	383	-2.39	0.0174
**Left superior longitudinal fasciculus**					
(Intercept)	-2.04	1.4	13.5	-1.45	0.17
Session	-0.113	0.0405	258	-2.79	0.00569
TV	5.44	3.79	13.6	1.43	0.174
fa_avg_lslf	-0.42	0.711	13.5	-0.591	0.565
wab_aq	0.00939	0.00246	13.4	3.81	0.00203
lesion_volume	2.23e-06	6.05e-06	13.4	0.369	0.718
Session:TV	0.398	0.11	258	3.62	3.59e-04
Session: fa_avg_lslf	0.00328	0.0197	257	0.167	0.868
Session:wab_aq	4.16e-05	6.55e-05	257	0.635	0.526
Session:lesion_volume	-5.46e-07	1.6e-07	257	-3.41	7.48e-04
**Left uncinate fasciculus**					

	Estimate	Std. Error	df	t value	Pr(>|t|)
(Intercept)	-1.82	1.87	6.46	-0.975	0.365
Session	-0.216	0.0475	165	-4.56	1.01e-05
TV	4.58	5.12	6.46	0.894	0.403
fa_avg_lunc	0.431	0.899	6.45	0.479	0.648
wab_aq	0.00545	0.00352	6.35	1.55	0.169
lesion_volume	4.35e-06	7.66e-06	6.35	0.568	0.59
Session:TV	0.745	0.128	165	5.82	3.06e-08
Session: fa_avg_lunc	-0.0108	0.022	165	-0.491	0.624
Session:wab_aq	-9.34e-05	6.68e-05	165	-1.4	0.164
Session:lesion_volume	-3.53e-07	1.45e-07	165	-2.43	0.0162
**Left arcuate fasciculus**					
(Intercept)	-3.35	2.05	3.49	-1.64	0.187
Session	-0.0711	0.0589	126	-1.21	0.229
TV	9.49	6.03	3.49	1.57	0.201
fa_avg_larc	-0.915	0.925	3.5	-0.988	0.386
wab_aq	0.0123	0.00497	3.55	2.49	0.0759
lesion_volume	-3.68e-06	6.69e-06	3.49	-0.549	0.616
Session:TV	0.241	0.175	126	1.38	0.171
Session: fa_avg_larc	0.0025	0.0273	126	0.0914	0.927
Session:wab_aq	1.27e-04	1.62e-04	126	0.781	0.436
Session:lesion_volume	-4.02e-07	1.92e-07	126	-2.1	0.0377
**Right inferior fronto-occipital fasciculus**					
(Intercept)	-1.51	0.965	27.9	-1.56	0.13
Session	-0.17	0.0261	464	-6.52	1.85e-10
TV	3.71	2.73	27.9	1.36	0.185
fa_avg_rifof	-0.0303	0.765	27.8	-0.0396	0.969
wab_aq	0.00814	0.00175	27.7	4.64	7.59e-05
lesion_volume	2.52e-06	4.04e-06	27.8	0.622	0.539
Session:TV	0.453	0.0735	464	6.16	1.6e-09
Session: fa_avg_rifof	0.0909	0.02	464	4.55	6.98e-06
Session:wab_aq	4.08e-05	4.5e-05	463	0.905	0.366
Session:lesion_volume	-6.46e-07	1.07e-07	464	-6.06	2.77e-09
**Right inferior longitudinal fasciculus**					
(Intercept)	-1.96	0.94	28.4	-2.09	0.0459

	Estimate	Std. Error	df	t value	Pr(>|t|)
Session	-0.195	0.0281	464	-6.93	1.42e-11
TV	3.96	2.53	28.4	1.57	0.128
fa_avg_rilf	0.993	0.819	28.2	1.21	0.236
wab_aq	0.00772	0.00156	28.2	4.96	3.06e-05
lesion_volume	9.31e-07	3.5e-06	28.3	0.266	0.792
Session:TV	0.517	0.0748	464	6.91	1.66e-11
Session: fa_avg_rilf	0.104	0.0226	463	4.59	5.78e-06
Session:wab_aq	6.86e-05	4.32e-05	463	1.59	0.113
Session:lesion_volume	-5.66e-07	9.92e-08	463	-5.71	2.05e-08
**Right superior longitudinal fasciculus**					
(Intercept)	-1.53	1.05	27.8	-1.47	0.154
Session	-0.155	0.0279	464	-5.56	4.47e-08
TV	3.78	2.81	27.8	1.34	0.19
fa_avg_rslf	-0.0171	0.668	28.2	-0.0256	0.98
wab_aq	0.00812	0.00176	27.8	4.62	7.97e-05
lesion_volume	2.42e-06	3.72e-06	27.8	0.651	0.52
Session:TV	0.463	0.0752	464	6.16	1.56e-09
Session: fa_avg_rslf	0.0348	0.02	464	1.74	0.083
Session:wab_aq	8.53e-05	4.59e-05	463	1.86	0.0639
Session:lesion_volume	-4.53e-07	9.79e-08	463	-4.63	4.85e-06
**Right uncinate fasciculus**					
(Intercept)	-2.11	1.01	27	-2.08	0.0472
Session	-0.137	0.0281	447	-4.87	1.56e-06
TV	3.98	2.8	27	1.42	0.167
fa_avg_runc	1.25	0.97	27	1.28	0.21
wab_aq	0.00767	0.00168	26.8	4.58	9.59e-05
lesion_volume	1.87e-06	3.59e-06	26.9	0.519	0.608
Session:TV	0.428	0.0793	447	5.4	1.09e-07
Session: fa_avg_runc	0.024	0.0277	448	0.866	0.387
Session:wab_aq	1.07e-04	4.43e-05	446	2.42	0.0161
Session:lesion_volume	-4.28e-07	9.77e-08	447	-4.38	1.45e-05
**Right arcuate fasciculus**					
(Intercept)	-1.4	1.04	23.4	-1.35	0.191
Session	-0.146	0.0281	395	-5.18	3.53e-07

	Estimate	Std. Error	df	t value	Pr(>|t|)
TV	4.13	2.99	23.4	1.38	0.18
fa_avg_rarc	-0.71	0.977	23.7	-0.727	0.475
wab_aq	0.00857	0.00226	23.4	3.79	9.27e-04
lesion_volume	4.49e-06	4.95e-06	23.5	0.909	0.373
Session:TV	0.418	0.0812	396	5.15	4.04e-07
Session: fa_avg_rarc	0.0252	0.0294	396	0.857	0.392
Session:wab_aq	2.3e-04	6.19e-05	396	3.72	2.29e-04
Session:lesion_volume	-2.46e-07	1.37e-07	396	-1.79	0.0734
**Mean of 12 white matter tracts**					
(Intercept)	-2.08	1.02	28.1	-2.04	0.0513
Session	-0.172	0.0285	464	-6.03	3.33e-09
TV	3.97	2.64	28.2	1.51	0.143
fa_mean	1.27	1.15	28	1.1	0.28
wab_aq	0.00731	0.00174	28	4.21	2.39e-04
lesion_volume	4.98e-07	3.83e-06	28.1	0.13	0.897
Session:TV	0.465	0.0746	464	6.22	1.08e-09
Session: fa_mean	0.0843	0.0301	463	2.8	0.00535
Session:wab_aq	6.59e-05	4.59e-05	463	1.43	0.152
Session:lesion_volume	-5.28e-07	1.03e-07	463	-5.11	4.65e-07

Supplementary Table 9. Linear mixed effects model results with mean functional connectivity of functional networks as covariates predicting naming accuracy. Higher TV remained significantly associated with greater naming accuracy improvement in all models.

	Estimate	Std. Error	df	t value	Pr(>|t|)
**Language network functional connectivity (LN_FC)**					
(Intercept)	-1.45	0.954	27.8	-1.52	0.139
Session	-0.139	0.0254	464	-5.46	7.81e-08
TV	3.28	2.93	27.8	1.12	0.272
LN_FC	1.22	2.01	27.9	0.609	0.547
wab_aq	0.00832	0.00173	27.7	4.8	4.93e-05
lesion_volume	3.04e-06	3.81e-06	27.8	0.796	0.433
Session:TV	0.486	0.0764	463	6.36	4.77e-10
Session: LN_FC	-0.119	0.0547	464	-2.18	0.0296
Session:wab_aq	9.19e-05	4.4e-05	463	2.09	0.0372

	Estimate	Std. Error	df	t value	Pr(>|t|)
Session:lesion_volume	-4.72e-07	9.88e-08	463	-4.78	2.38e-06
**Salience network functional connectivity (SN_FC)**					
(Intercept)	-1.89	0.96	27.9	-1.97	0.0594
Session	-0.115	0.0259	464	-4.44	1.15e-05
TV	5.54	3	27.9	1.85	0.0752
SN_FC	-1.38	0.891	27.8	-1.54	0.134
wab_aq	0.00783	0.00168	27.7	4.68	6.87e-05
lesion_volume	2.79e-07	3.86e-06	27.8	0.0723	0.943
Session:TV	0.349	0.0801	464	4.35	1.66e-05
Session: SN_FC	0.0804	0.0236	464	3.41	6.99e-04
Session:wab_aq	1.28e-04	4.27e-05	463	2.99	0.00289
Session:lesion_volume	-2.92e-07	1.01e-07	464	-2.9	0.00392
**Dorsal attention network functional connectivity (DAN_FC)**					
(Intercept)	-1.48	0.916	28.1	-1.61	0.118
Session	-0.134	0.0256	464	-5.22	2.72e-07
TV	3.97	2.72	28	1.46	0.155
DAN_FC	-0.453	0.433	28.1	-1.04	0.305
wab_aq	0.00781	0.00166	27.9	4.7	6.43e-05
lesion_volume	6.3e-07	3.92e-06	27.9	0.161	0.874
Session:TV	0.452	0.0751	464	6.02	3.51e-09
Session: DAN_FC	-0.00781	0.0122	464	-0.641	0.522
Session:wab_aq	1.1e-04	4.34e-05	463	2.54	0.0115
Session:lesion_volume	-4.39e-07	1.04e-07	463	-4.23	2.82e-05
**Default mode network functional connectivity (DMN_FC)**					
(Intercept)	-1.56	0.971	27.9	-1.6	0.12
Session	-0.119	0.0264	464	-4.52	7.77e-06
TV	3.8	2.82	27.9	1.35	0.188
DMN_FC	0.0601	0.603	27.8	0.0996	0.921
wab_aq	0.00811	0.00169	27.7	4.79	5.06e-05
lesion_volume	2.46e-06	3.66e-06	27.8	0.673	0.507

	Estimate	Std. Error	df	t value	Pr(>|t|)
Session:TV	0.42	0.0761	464	5.52	5.77e-08
Session: DMN_FC	-0.0338	0.0155	463	-2.17	0.0302
Session:wab_aq	1.06e-04	4.29e-05	463	2.46	0.0141
Session:lesion_volume	-4.34e-07	9.55e-08	463	-4.55	6.96e-06

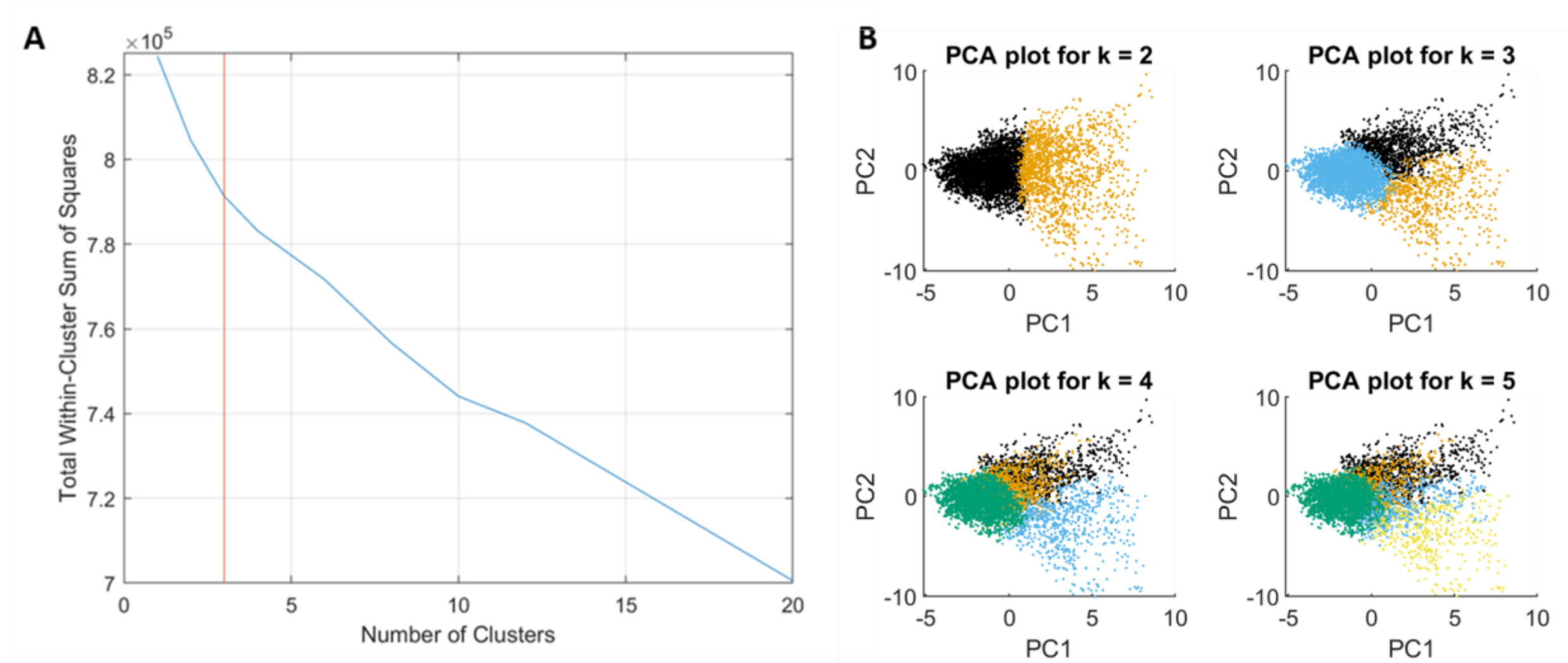

Supplementary Figure 1. Determination of k value for k-means clustering of language network dFC time windows. **A.** Elbow plot with vertical red line marking selected k value. **B.** Principal components analysis was used to reduce dimensionality for data visualization. Plots correspond to the first two principal components. Each data point corresponds to one time window for one participant (N = 30 subjects × 164 time windows = 4920) and marker color indicates the cluster a time window was assigned to using the respective k value. Because k values of 4 or greater produced clusters with significant overlap, a k value of 3 was used to cluster dFC time windows into group-level states.

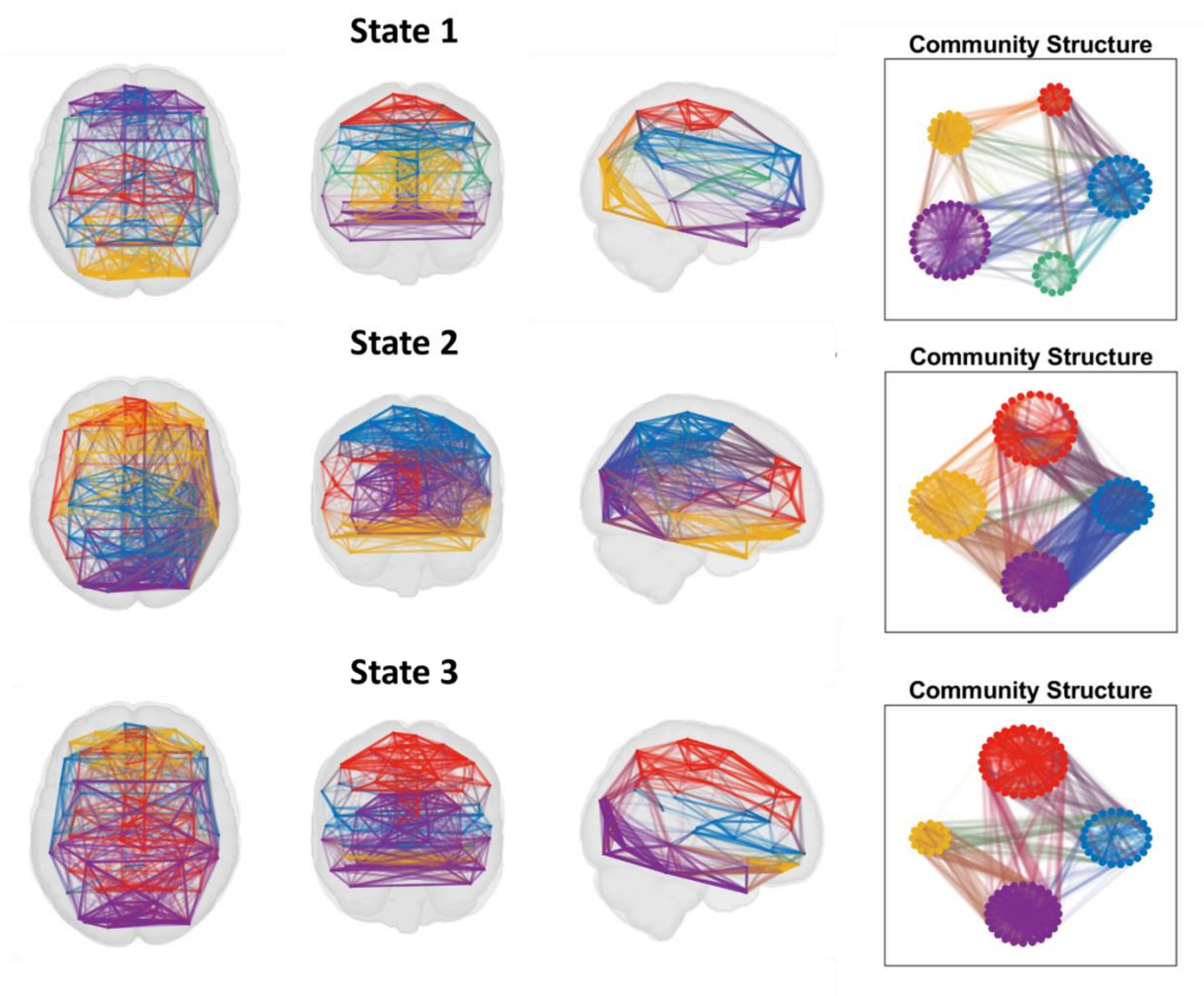

Supplementary Figure 2. Whole cortex dFC per state computed as mean dFC of clustered time windows. Colors indicate communities identified using a Louvain community detection algorithm. Panels on the right show nodes organized according to this community structure

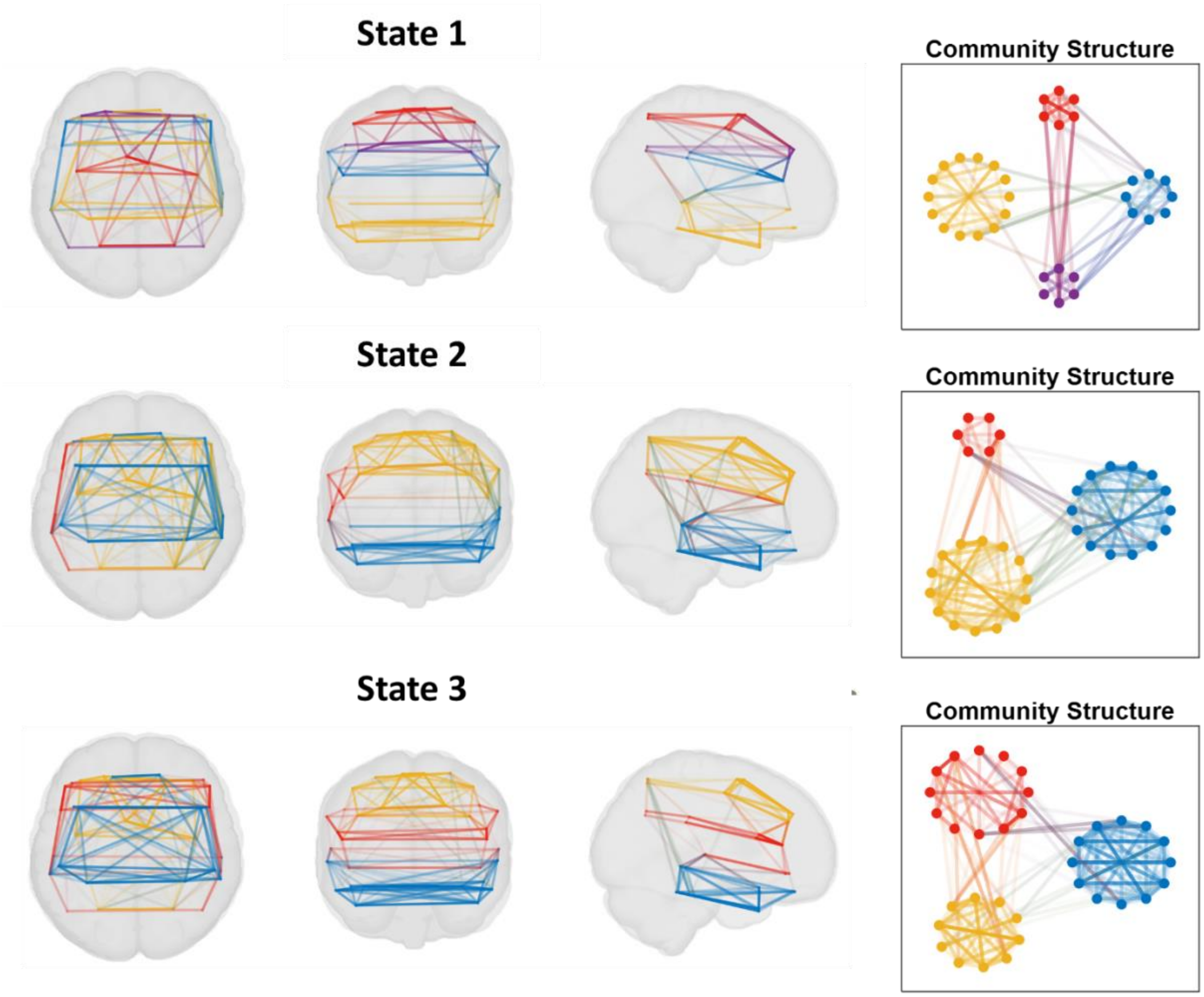

Supplementary Figure 3. Language network dFC per state computed as mean dFC of clustered time windows. Colors indicate communities identified using a Louvain community detection algorithm. Panels on the right show nodes organized according to this community structure

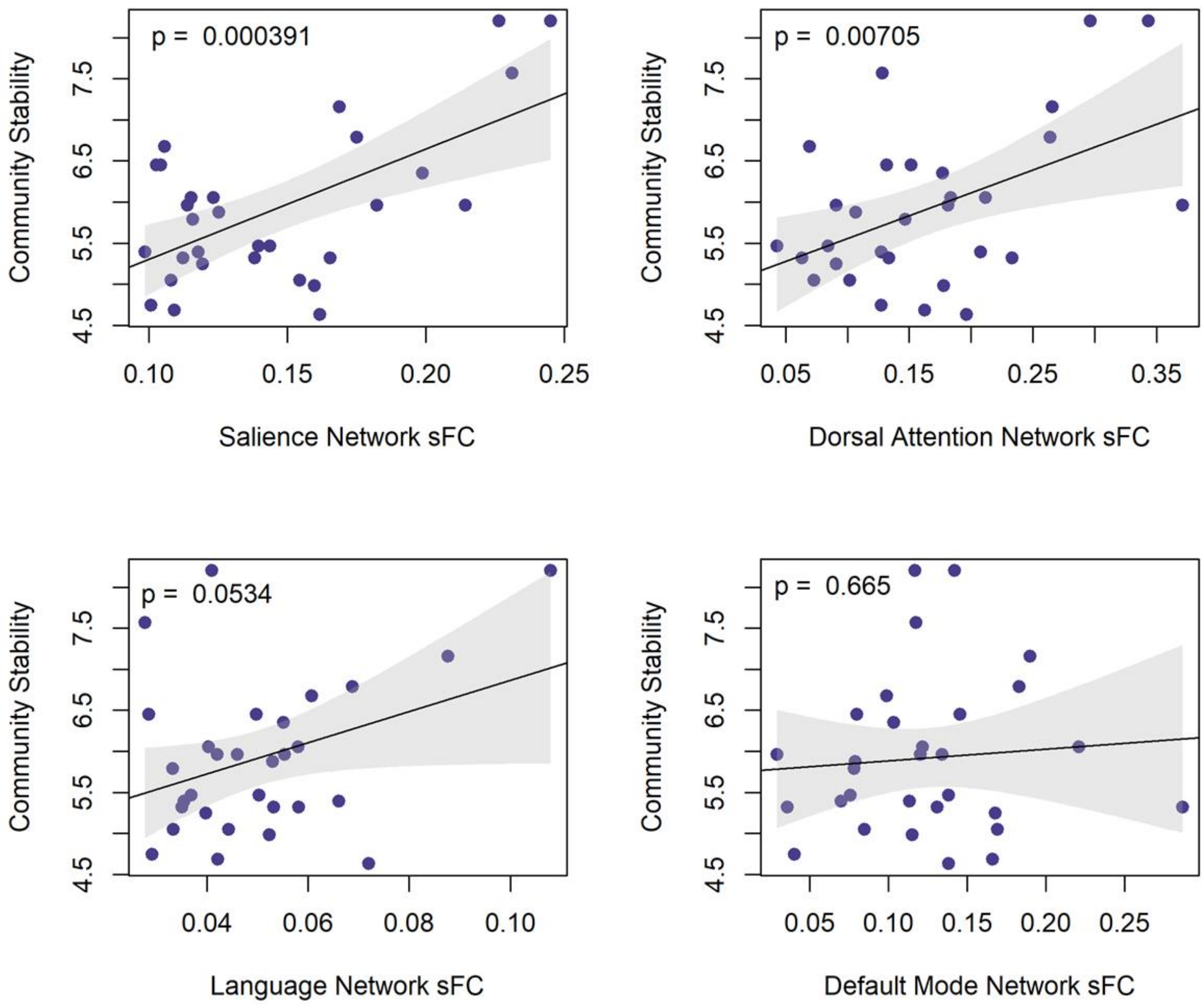

Supplementary Figure 4. Plots of community stability as a function of static functional connectivity (sFC) in four networks. The reported p-values are from Pearson's correlation tests (N=30). Each data point represents one participant.
